# *Rickettsia tillamookensis*-like strain in brown dog ticks in Brazil

**DOI:** 10.1186/s13071-026-07290-8

**Published:** 2026-02-19

**Authors:** Lucas Lisboa Nunes Bonifácio, Kamila Gaudêncio da Silva Sales, Ennya Rafaella Neves Cardoso, Felipe da Silva Krawczak, Filipe Dantas-Torres

**Affiliations:** 1 Aggeu Magalhães Institute, Oswaldo Cruz Foundation (Fiocruz), Recife, Pernambuco Brazil; 2 Federal University of Goiás, Goiânia, Brazil

**Keywords:** *Rickettsia tillamookensis*-like, Ticks, *Rhipicephalus linnaei*, Phylogeny

## Abstract

Brown dog ticks (*Rhipicephalus linnaei*) collected from dogs in northeastern Brazil in 2015 tested positive for a rickettsial organism phylogenetically related to *Rickettsia tillamookensis* (referred to as *R. tillamookensis*-like). One of the dogs with *R. tillamookensis*-like-positive ticks tested positive for IgG antibodies to *Rickettsia rickettsii* antigens, suggesting a potential reaction to spotted fever group rickettsiae. Future research should assess whether the rickettsia detected herein is *R. tillamookensis* or a closely related species, its distribution, potential vectors, and possible pathogenicity to humans.

**Graphical Abstract:**

**Figure:**
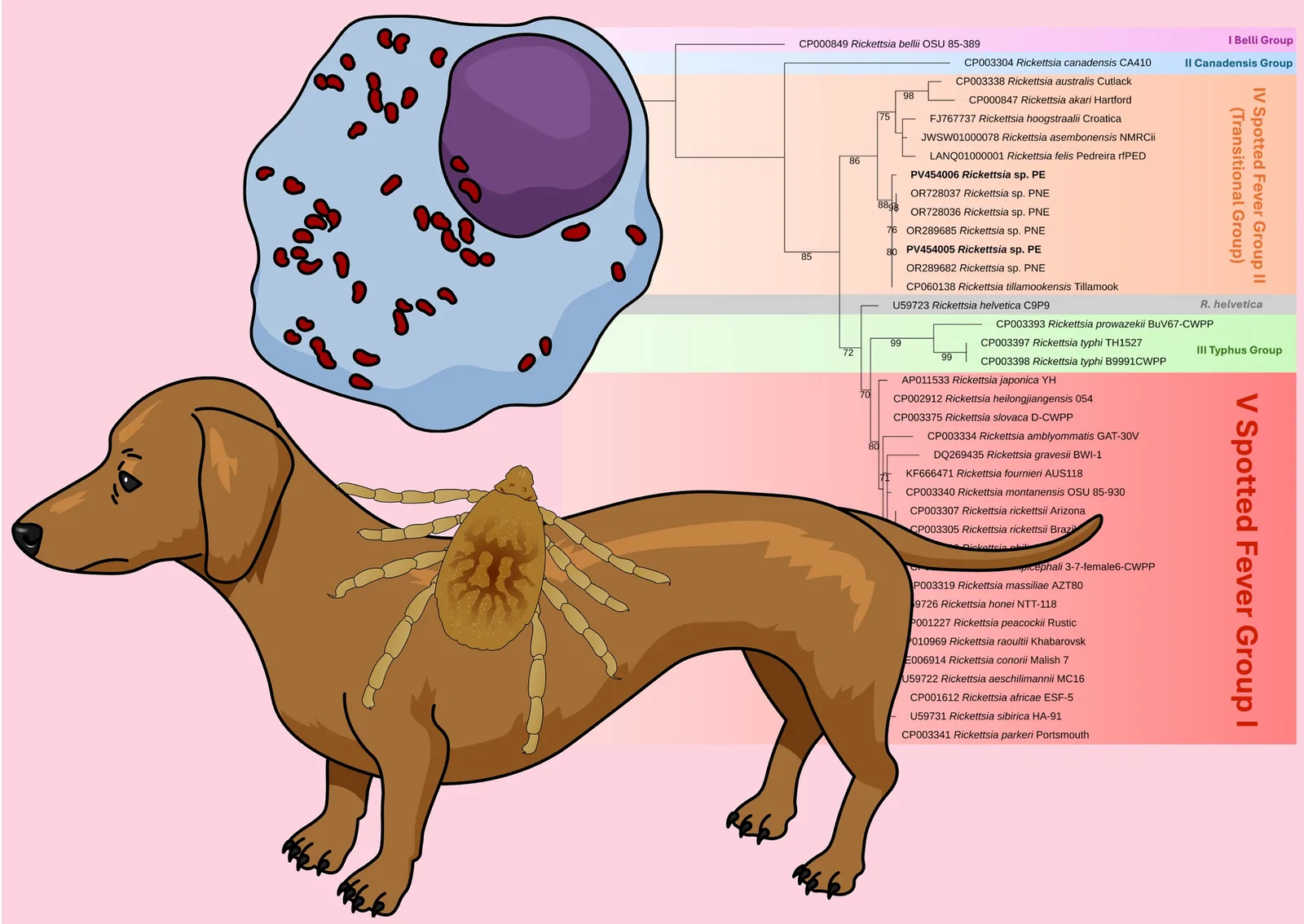

Ticks are important vectors for significant pathogens affecting humans and animals. For example, tick-borne rickettsiae are found worldwide, with new species being discovered sporadically. One such species, *Rickettsia tillamookensis*, was initially isolated from the western black-legged tick (*Ixodes pacificus*) collected in 1967 in Tillamook County, Oregon, USA, and recently (2021) formally described [1]. We report the detection of a rickettsia phylogenetically related to *R. tillamookensis* (referred to from now on as *R. tillamookensis*-like) in brown dog ticks (*Rhipicephalus linnaei*) collected from dogs in Brazil.

Ticks (*n* = 373) were collected from 100 privately owned dogs in Goiana, Pernambuco state, northeastern Brazil, during a previous study conducted in 2015–2016 [2]. In brief, ticks preserved in 70% ethanol were morphologically identified [3] and then grouped into pools of two to five ticks per dog. Pooled prevalence and 95% confidence limits (95% CL) were estimated using Epitools (https://epitools.ausvet.com.au/ppvariablepoolsize).

Genomic DNA was extracted from the ticks using Chelex^®^ 100 resin (Bio-Rad Laboratories, Hercules, CA, USA). Partial fragments of the citrate synthase (*gltA*) gene of *Rickettsia* spp. and the 16S rRNA gene of ticks were amplified via polymerase chain reactin (PCR) as previously described [4, 5]. PCR assays were conducted on a Veriti^®^ 96-well thermocycler (Applied Biosystems, Foster City, CA, USA), and the results were analyzed by electrophoresis on a 1.5% agarose gel stained with ethidium bromide under ultraviolet (UV) illumination. The PCR products were purified using the PureLink™ PCR Micro Kit (Invitrogen, Carlsbad, CA, USA) and sequenced in both directions with the same primers as for PCR and the BigDye Terminator v3.1 sequencing kit (Applied Biosystems, Foster City, CA, USA) on an ABI Prism 3500XL (Applied Biosystems, Foster City, CA, USA). Sequences were trimmed (Phred ≥ 20) and assembled using Clustal Omega in Geneious Prime v. 2025.0.3 (https://www.geneious.com). The resulting consensus sequences underwent similarity searches using the Basic Local Alignment Search Tool (BLAST) for nucleotides (http://blast.ncbi.nlm.nih.gov/Blast.cgi). The consensus *gltA* gene sequences generated in this study were aligned with sequences retrieved from GenBank using ClustalW in MEGA11 [6, 7]. The evolutionary history of *Rickettsia* spp. was inferred using maximum likelihood with 1000 ultrafast bootstrap replicates, on the basis of a dataset from a previous study [8]. Phylogenetic analysis was performed with W-IQ-TREE [9], with the best-fit evolutionary model (K3Pu + F + G4) determined using ModelFinder. The obtained consensus tree was rooted with a *Rickettsia bellii* sequence (GenBank accession no. CP00084). The final phylogenetic tree was edited using iTOL v7 (https://itol.embl.de). We also assessed the genetic distance between our sequences and related species using the Kimura 2-parameter model in MEGA11.

Dogs underwent serological testing using an immunofluorescence assay (IFA) targeting antibodies to four *Rickettsia* spp. antigens from Brazil (*R. rickettsii* strain Pampulha, *Rickettsia parkeri* strain Atlantic rainforest, *Rickettsia amblyommatis* strain Ac37, and *R. bellii* strain Mogi), as described in prior studies [10–12].

Ticks were morphologically identified as 220 females and 153 males of *R. linnaei*. Our representative 16S rRNA gene sequence of *R. linnaei* (GenBank accession no. PV449820) showed 100% (434/434) identity with *R. linnaei* from the southwestern USA (GenBank accession no. OM985256).

Four pools of *R. linnaei* (nine females and six males) tested positive for the *gltA* gene, with an estimated pooled prevalence of 1.1% (95% CL: 0.3–2.5%). Sequences from two pools of *R. linnaei* containing two ticks (one male and one female) and five ticks (three males and two females) showed 99.2% (387/390; GenBank accession no. PV454006) and 99.7% (382/383; GenBank accession no. PV454005) identity with the *R. tillamookensis* type strain (Tillamook 23) (GenBank accession no. CP060138). Attempts to sequence the PCR products from other positive samples were unsuccessful, likely owing to the low DNA amount. We also attempted to amplify other rickettsial genes, such as *ompB*, but were unsuccessful.

Our partial *gltA* gene sequences clustered with *R. tillamookensis* and related sequences previously reported in Brazil within spotted fever group II (Fig. 1). The pairwise distance between *R. tillamookensis* and our sequences ranged from 0.003 to 0.005 (Table 1). This distance is less than that (0.011–0.016) observed between the nearest species within the spotted fever group II, namely, *Rickettsia felis*, *Rickettsia asembonensis*, and *Rickettsia hoogstraalii*. This suggests that the species detected in this study is, in fact, *R. tillamookensis*. Still, we prefer to wait for further research with additional sequences and genetic markers to confirm this hypothesis.

**Fig. 1:**
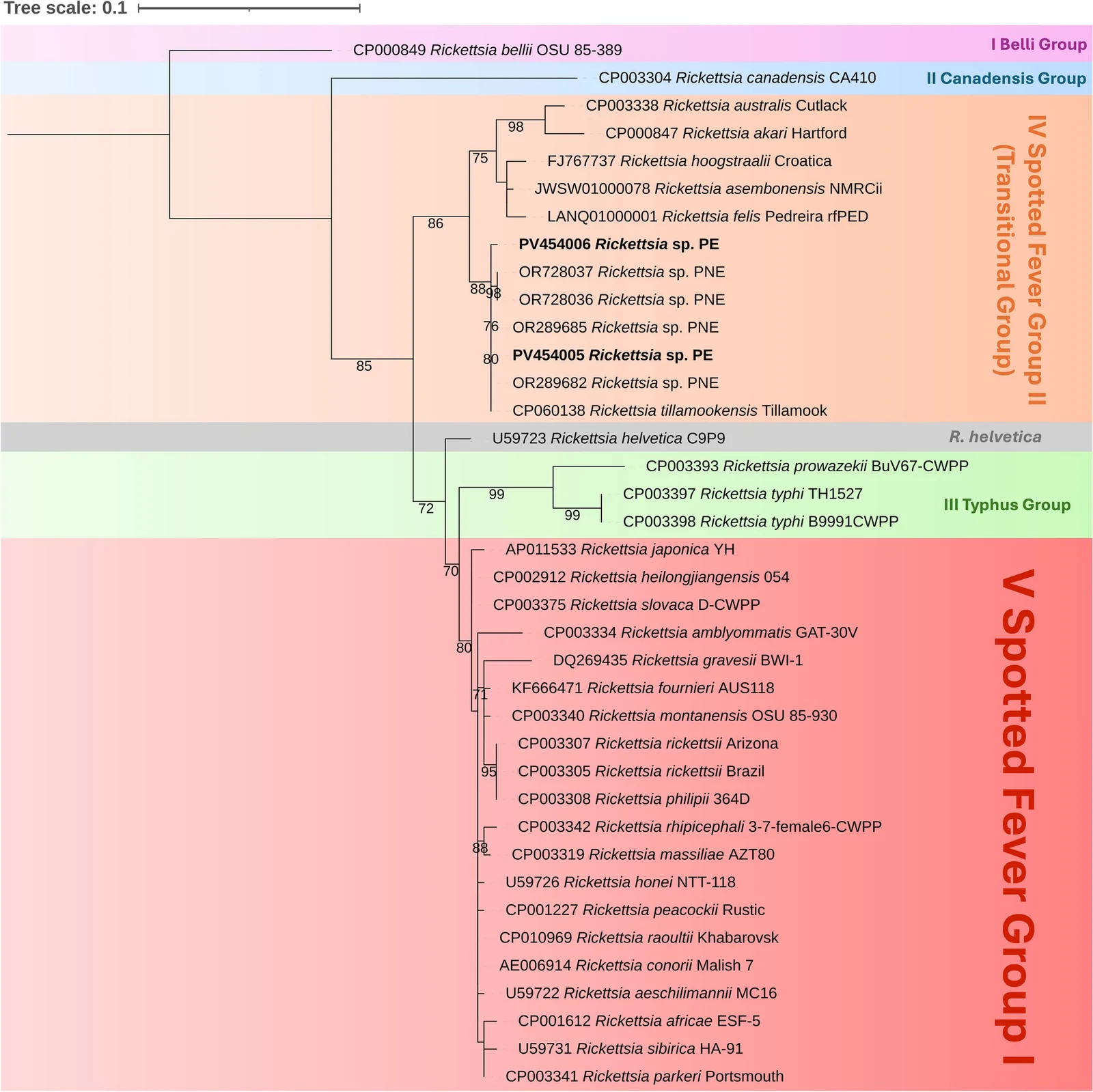
Phylogenetic tree reconstruction of the *Rickettsia* genus based on a fragment of the *gltA* gene, utilizing a dataset of 38 sequences (30 species) of 350 base pairs (bp). The tree was inferred using the maximum-likelihood method with 1000 ultrafast bootstrap replicates (bootstrap values < 70 were omitted) and the K3Pu + F + G4 substitution model. *Rickettsia bellii* (CP00084) served as the outgroup. Sequences generated in this study are highlighted in bold

**Table 1:** Estimates of evolutionary divergence between the *gltA* gene sequences of *Rickettsia* spp. belonging to the spotted fever group II (ancestral group)

	1	2	3	4	5	6	7	8
1. CP060138 *Rickettsia tillamookensis* strain Tillamook 23	–							
2. PV454006 *Rickettsia* sp. strain PE87	**0.005**							
3. PV454005 *Rickettsia* sp. strain PE07	**0.003**	**0.003**						
4. LANQ01000001 *Rickettsia felis strain* Pedreira rfPED	0.030	0.036	0.033					
5. JWSW01000078 *Rickettsia asembonensis* strain NMRCii	0.027	0.033	0.030	0.011				
6. FJ767737 *Rickettsia hoogstraalii* strain Croatica	0.033	0.039	0.036	0.016	0.011			
7. CP000847 *Rickettsia akari* strain Hartford	0.051	0.057	0.054	0.051	0.045	0.045		
8. CP003338 *Rickettsia australis* strain Cutlack	0.051	0.057	0.054	0.045	0.039	0.039	0.027	–

The number of base substitutions per site between sequences is presented. Analyses were conducted using the Kimura 2-parameter model. This analysis included eight nucleotide sequences, and all positions containing gaps and missing data were eliminated (complete deletion option). There were 383 positions in the final dataset. The distances between the *Rickettsia tillamookensis* strain Tillamook 23 and our sequences are in bold

In the serological assessment, 43% of the dogs tested positive for antibodies against *Rickettsia* spp. Specifically, 4 dogs showed reactions to *R. rickettsii* antigens (with titers ranging from 64 to 512), while 43 reacted to *R. bellii* (with titers ranging from 64 to 2048). None of the dogs were positive to *R. parkeri* or *R. amblyommatis* antigens.

Our data indicate that *R. tillamookensis*, or a closely related rickettsia, circulates among brown dog ticks and possibly among dogs in northeastern Brazil. A high level of exposure (43%) to rickettsial antigens was detected among dogs from which ticks were collected. While we did not test the dogs against *R. tillamookensis* antigens, we cannot rule out that at least some reactions were to *R. tillamookensis* or another species from the ancestral group. In addition to the original report in *I. pacificus* from Tillamook County, Oregon [1], this rickettsia has also been documented in *I. pacificus* from California [13, 14] and *Ixodes scapularis* from Oklahoma [15]. More recently, strains closely related to *R. tillamookensis* have also been detected in *Amblyomma sculptum* and *Amblyomma triste* from central-western Brazil [8] and in *Haemaphysalis inermis* from Bulgaria [16]. Further research is needed to establish whether this rickettsia is a strain of *R. tillamookensis* or a closely related but different species. Our attempts to amplify and sequence other genes (e.g., outer membrane protein B gene) were unsuccessful, likely owing to the low amount of DNA available in the samples.

*Rickettsia tillamookensis* belongs to the spotted fever group II (the transitional group), indicating an ancestral relationship with the rickettsiae in this group [1, 8]. Other rickettsiae belonging to the spotted fever group II have been reported in *R. sanguineus*, including *Rickettsia asemboensis* [17].

One of the dogs with *R. tillamookensis*-like-positive ticks showed anti-*R. rickettsii* antibodies (titer = 128), suggesting previous exposure to spotted fever group rickettsiae. However, this cannot be confirmed, as the dog also reacted to *R. bellii* antigens.

The pathogenicity of *R. tillamookensis* in humans remains unknown; however, experimentally infected guinea pigs exhibited mild clinical signs, including low fever and slight scrotal edema [1]. Detecting a rickettsia related to *R. tillamookensis* in a widely distributed urban tick in Brazil suggests that its distribution throughout the country may be broader than currently recognized. Indeed, *R. linnaei* is a vector for many significant pathogens [18]. For instance, it is a confirmed vector of *R. rickettsii* and a suspected vector of *Rickettsia massiliae* in Mexico [19]. Unfortunately, attempts to amplify other gene fragments were unsuccessful, which constitutes the main limitation of the present work. Therefore, future studies are essential to assess the identity of this *R. tillamookensis*-like strain in Brazil, its distribution, potential vectors, and potential pathogenicity in humans.

## Data Availability

The DNA sequences generated in this study have been deposited in GenBank (accession nos. PV454005, PV454006, PV449820).

## References

[1] Gauthier DT, Karpathy SE, Grizzard SL, Batra D, Rowe LA, Paddock CD. Characterization of a novel transitional group *Rickettsia* species (*Rickettsia tillamookensis* sp. nov.) from the western black-legged tick, *Ixodes pacificus*. Int J Syst Evol Microbiol. 2021;71:004880. 34214027 10.1099/ijsem.0.004880 PMC8489840

[2] Dantas-Torres F, Figueredo LA, Sales KGDS, Miranda DEO, Alexandre JLA, da Silva YY, et al. Prevalence and incidence of vector-borne pathogens in unprotected dogs in two Brazilian regions. Parasit Vect. 2020;13:195. 10.1186/s13071-020-04056-8 PMC7171771 32312297

[3] Dantas-Torres F, Martins TF, Muñoz-Leal S, Onofrio VC, Barros-Battesti DM. Ticks (Ixodida: Argasidae, Ixodidae) of Brazil: updated species checklist and taxonomic keys. Ticks Tick Borne Dis. 2019;10:101252. 31255534 10.1016/j.ttbdis.2019.06.012

[4] Labruna MB, McBride JW, Bouyer DH, Camargo LM, Camargo EP, Walker DH. Molecular evidence for a spotted fever group *Rickettsia* species in the tick *Amblyomma longirostre* in Brazil. J Med Entomol. 2004;41:533–7. 15185961 10.1603/0022-2585-41.3.533

[5] Mangold AJ, Bargues MD, Mas-Coma S. Mitochondrial 16S rDNA sequences and phylogenetic relationships of species of *Rhipicephalus* and other tick genera among Metastriata (Acari: Ixodidae). Parasitol Res. 1998;84:478–84. 9660138 10.1007/s004360050433

[6] Thompson JD, Higgins DG, Gibson TJ. CLUSTAL w: improving the sensitivity of progressive multiple sequence alignment through sequence weighting, position-specific gap penalties and weight matrix choice. Nucleic Acids Res. 1994;22:4673–80. 7984417 10.1093/nar/22.22.4673 PMC308517

[7] Tamura K, Stecher G, Kumar S. MEGA11: molecular evolutionary genetics analysis version 11. Mol Biol Evol. 2021;38:3022–7. 33892491 10.1093/molbev/msab120 PMC8233496

[8] Paludo RLDR, Paula WVF, Neves LC, de Paula LGF, de Lima NJ, da Silva BBF, et al. Rickettsial infection in ticks from a national park in the Cerrado biome, midwestern Brazil. Pathogens. 2023;13:13. 38251322 10.3390/pathogens13010013 PMC10818336

[9] Trifinopoulos J, Nguyen LT, von Haeseler A, Minh BQ. W-IQ-TREE: a fast online phylogenetic tool for maximum likelihood analysis. Nucleic Acids Res. 2016;44:W232–5. 27084950 10.1093/nar/gkw256 PMC4987875

[10] Horta MC, Labruna MB, Sangioni LA, Vianna MC, Gennari SM, Galvão MA, et al. Prevalence of antibodies to spotted fever group rickettsiae in humans and domestic animals in a Brazilian spotted fever-endemic area in the state of São Paulo, Brazil: serologic evidence for infection by *Rickettsia rickettsii* and another spotted fever group *Rickettsia*. Am J Trop Med Hyg. 2004;71:93–7. 15238696

[11] Labruna MB, Horta MC, Aguiar DM, Cavalcante GT, Pinter A, Gennari SM, et al. Prevalence of *Rickettsia* infection in dogs from the urban and rural areas of Monte Negro municipality, western Amazon, Brazil. Vector Borne Zoonotic Dis. 2007;7:249–55. 17627445 10.1089/vbz.2006.0621

[12] Neves LC, Paula WVF, de Paula LGF, da Silva BBF, Dias SA, Pereira BG, et al. Detection of *Rickettsia* spp. in animals and ticks in midwestern Brazil, where human cases of rickettsiosis were reported. Animals. 2023;13:1288. 37106851 10.3390/ani13081288 PMC10135036

[13] Paddock CD, Slater K, Swei A, Zambrano ML, Kleinjan JE, Padgett KA, et al. Detection and isolation of *Rickettsia tillamookensis* (Rickettsiales: Rickettsiaceae) from *Ixodes pacificus* (Acari: Ixodidae) from multiple regions of California. J Med Entomol. 2022;59:1404–12. 35468215 10.1093/jme/tjac038

[14] Trent E, Swei A, Feiszli T, Saunders MEM, Zhong J. Prevalence of *Rickettsia* species phylotype G022 and *Rickettsia tillamookensis* in *Ixodes pacificus* nymphs and adults from Northern California. Ticks Tick Borne Dis. 2025;16:102463. 40112617 10.1016/j.ttbdis.2025.102463 PMC12232624

[15] Noden BH, Gilliland M, Propst J, Slater K, Karpathy SE, Paddock CD. *Rickettsia tillamookensis* (Rickettsiales: Rickettsiaceae) in *Ixodes scapularis* (Acari: Ixodidae) in Oklahoma. J Med Entomol. 2024;61:257–60. 37738127 10.1093/jme/tjad133

[16] Polsomboon Nelson S, Ergunay K, Bourke BP, et al. Nanopore-based metagenomics reveal a new *Rickettsia* in Europe. Ticks Tick Borne Dis. 2024;15:102305. 38150911 10.1016/j.ttbdis.2023.102305

[17] Dall’Agnol B, Souza U, Webster A, Weck B, Stenzel B, Labruna M, et al. Candidatus Rickettsia asemboensis in *Rhipicephalus sanguineus* ticks. Brazil Acta Trop. 2017;167:18–20. 27986544 10.1016/j.actatropica.2016.12.008

[18] Dantas-Torres F, de Sousa-Paula LC, Otranto D. The *Rhipicephalus sanguineus* group: updated list of species, geographical distribution, and vector competence. Parasit Vect. 2024;17:540. 10.1186/s13071-024-06572-3 PMC11681662 39731169

[19] Nieto-Cabrales JF, Salceda-Sánchez B, Zazueta-Islas HM, et al. New records of *Rhipicephalus linnaei* infected by *Rickettsia massiliae* from Central Mexico. Zoonoses Public Health. 2024;71:217–24. 38050875 10.1111/zph.13101

